# Psychosis in systemic lupus erythematosus (SLE): 40-year experience of a specialist centre

**DOI:** 10.1093/rheumatology/keab160

**Published:** 2021-02-25

**Authors:** Esha Abrol, Ester Coutinho, Michael Chou, Melanie Hart, Angela Vincent, Robert Howard, Michael S Zandi, David Isenberg

**Affiliations:** 1 Division of Psychiatry, University College London; 2 Department of Basic and Clinical Neuroscience, Institute of Psychiatry Psychology and Neuroscience, King’s College London; 3 Neuroimmunology Laboratory, National Hospital for Neurology and Neurosurgery, London; 4 Nuffield Department of Clinical Neurosciences, University of Oxford, Oxford; 5 Department of Neuromuscular Diseases, UCL Queen Square Institute of Neurology; 6 Centre for Rheumatology, Division of Medicine, University College London (UCL), London, UK

**Keywords:** lupus, psychosis, autoimmune psychosis, SLE

## Abstract

**Objectives:**

The long-term outcome of psychosis in association with systemic lupus erythematosus (SLE) has been insufficiently characterised. We used a specialist centre cohort of patients with SLE and psychosis to investigate their clinical outcome and phenotypic and laboratory characteristics.

**Methods:**

Retrospective cohort study of 709 SLE patients seen at a specialist centre between January 1978 and November 2018. Clinical, biochemical and immunological characteristics (Bonferroni corrected), and serum neuronal surface antibody profile using novel cell-based assays, were compared between patients with and without psychosis.

**Results:**

Eighteen (18/709, 2.5%) patients developed lupus psychosis over a mean ± SD of 17.5 ± 11.0 years follow-up. Psychosis fully remitted in 66.7% (12/18) with a combination of antipsychotic (in 38.9%) and immunosuppressive therapy (methylprednisolone 72.2%, cyclophosphamide 55.6%, rituximab 16.7%, plasma exchange 27.8%, prednisolone 50%). Patients who developed lupus psychosis may be more likely to have anti-RNP antibodies (50.0% *vs* 26.5%) and less likely to have anti-cardiolipin antibodies (5.6% *vs* 30.0%), but this was not significant in our small sample. Neuronal surface autoantibody tests found GABA_B_R autoantibodies in 3/10 (30.0%) lupus psychosis patients compared with only 3/27 (11.1%) in age- and sex-matched SLE controls using fixed cell-based assays (*P* =0.114). However, GABA_B_R antibodies were not replicated using a live cell-based assay. NMDAR-antibodies were not detected with fixed or live cell assays in any samples.

**Conclusion:**

Lupus psychosis is rare but treatable. In this rare sample of eighteen patients from a 40-year cohort, no significant biomarker was found, but some preliminary associations warrant further exploration in a larger multicentre analysis.

**Rheumatology key messages**

- Lupus psychosis is rare and highly responsive to treatment with a good prognosis.
- In this preliminary study, no significant serological or neuronal surface autoantibody biomarker was found.
- However, a possible autoantibody mediated mechanism in at least some patients requires further exploration.

## Introduction

Neuropsychiatric manifestations of SLE (NPSLE) include diffuse, focal, psychiatric, central, peripheral and autonomic nervous system disorders due to primary SLE disease [1]. NPSLE have been classified by the American College of Rheumatology (ACR) into 19 syndromes [2]. Unanimously, NPSLE are associated with a significant morbidity, reduction in patient-reported health-related quality of life [3] and increased mortality [4].

Within NPSLE, psychiatric syndromes are present in at least half of SLE patients, most commonly mood disorders (depression and anxiety; 3.6% to 75%) [5]. However, it has been suggested that other psychiatric features such as psychosis are underreported [6]. Lupus psychosis is a distinct immunologically driven psychosis occurring in patients with SLE after excluding primary psychotic disorder, substance- or drug-induced psychotic disorder, metabolic conditions or psychological mediated reactions to SLE [2]. Despite being included in the original ACR criteria in 1971 [1], little is known about this form of psychosis. Similar to all psychiatric manifestations in SLE, the reported prevalence of lupus psychosis ranges wildly, from 1.9–29.8% depending on the criteria used and duration of follow-up [7, 8]. Of the various antibodies reported in SLE, at least 20 (∼11 brain-specific and 9 systemic), have been inconsistently associated with both NPSLE and lupus psychosis [9, 10]. One of these is anti-ribosomal P antibodies, which despite showing initial promise as a sensitive biomarker for lupus psychosis [11], is likely to be non-specific [12]. Antibodies to the N-methyl-D-aspartate (NMDA) receptor, resulting from a cross-reaction with double-stranded DNA (dsDNA) represented another plausible target in lupus psychosis. Some studies have found antibodies to the NMDAR short peptide (DWEYS) NR2 on ELISA to be associated with diffuse neuropsychiatric events such as depression, poor learning and memory, and paranoia [13]. However, a meta-analysis showed that DWEYS positivity was present in as many as 30% of all SLE patients and not associated with neuropsychiatric symptoms [9]. Moreover, it is now accepted that the most appropriate method for detecting antibodies that might be causative is cell-based assay [14]. NMDAR and other autoantibodies have not been found in some SLE cohorts [15]. Comprehensive evaluation of patients who develop psychosis in SLE could guide future care and contribute to our understanding of the immunological basis of psychosis.

The present study aimed to identify SLE psychosis from a large case series of SLE patients followed up at a single specialist centre for over 40 years and to assess clinical and immunological characteristics. We tested serum from SLE psychosis patients for an array of antibodies directed against neuronal cell surface antigens that have been implicated as targets in psychosis (GABA_B_R, DPPX, AMPAR1/2, NMDAR, LGI1, CASPR2) [14] in order to try and identify potential biomarkers. This cohort has been previously reported in 2008; however, it now boasts increased numbers, extended duration of follow-up, more detailed psychiatric features and neuronal autoantibody testing [16].

## Methods

### Study design and participants

University College London Hospital (UCLH) NHS Foundation Trust has followed up 709 patients with SLE during a 40-year censorship period (January 1978 to November 2018). All patients met at least four of the revised American College of Rheumatology (ACR) classification criteria for SLE [17]. Clinical histories were examined to verify the diagnosis of lupus psychosis, as per the 1999 ACR NPSLE nomenclature and case definitions for psychosis [2]. All patients had at least 12 months follow-up after the diagnosis of psychosis. Patients who were lost to follow-up or who had died were censored at the last clinic visit.

### Procedures

#### Clinical, biochemical and immunological characteristics

Patient data were retrospectively collected from medical and psychiatric clinical records at UCLH. Detailed clinical information was collected at every outpatient appointment and every inpatient admission and stored on electronic and paper databases. This forms part of the British Isles Lupus Assessment Group (BILAG) index; a reliable and valid scoring system for assessing clinical disease activity in SLE [18]. Both BILAG and the updated version, BILAG 2004, capture CNS manifestations in a comprehensive manner. All patients with symptomatic psychosis were assessed by a psychiatrist, as recommended by ACR [2]. Gender, ethnicity, age at diagnosis of SLE, duration of follow up, death and/or loss-to-follow up were assessed, as well as age at psychotic episode for patients with lupus psychosis. Data on the features of SLE, NPSLE and routine serological data were collected as per ACR recommendations (Table 1). 

**Table 1:** Method of determination for serological tests in SLE

Routine serological test	Method of determination
Anti-nuclear antibodies (ANA)	Indirect immunofluorescence. ANAs were considered positive if the titre was >1/80. Anti-double-stranded DNA (dsDNA) antibodies were measured by standard ELISA and defined as positive if more than twice the upper limit of normal (50 IU/mL on three occasions) or if positive by a *Crithidia luciliae* test.
Anti-cardiolipin (aCL) antibodies	Anti-cardiolipin was determined by ELISA and results were considered positive if medium-to-high titres (>20 IgG phospholipid units or IgM phospholipid units) were present on two or more occasions at least 6 weeks apart.
Lupus anticoagulant (LA)	Lupus anticoagulant activity was detected by coagulation assays (dilute Russell’s viper venom time) according to the guidelines of the International Society on Thrombosis and Hemostasis.
Anti-RNP antibodies	All by standard ELISA.
Anti-Ro/La antibodies	
Anti-Sm antibodies	
Anti-Ribosomal P antibodies	
Rheumatoid Factor (RF)	Sheep cell agglutination. Rheumatoid factor was considered positive if the titre was >1/80.
C3 count	Laser nephelometer

For patients with lupus psychosis, the investigation variables (normal/abnormal EEG, normal/abnormal MRI, normal/abnormal brain perfusion scan, normal/abnormal CSF examination) and treatment variables (immunosuppressive therapy for induction of remission, immunosuppressive therapy for maintenance of remission, psychiatric treatment) were collected. Treatment with prednisolone was divided into low (0–7.5 mg/day), medium (7.5–19 mg/day) and high (≥20 mg/day) dose. For patients with lupus psychosis, the short (six months after the initial first episode of psychosis) and long-term (one year and beyond) outcome of psychosis was established, as guided by previous literature [7, 19].

#### Fixed cell-based assays

Serum samples of lupus psychosis patients were tested at the Neuroimmunology and CSF laboratory, National Hospital for Neurology and Neurosurgery, Queen Square (London, UK) by E.A. and M.C. using a multiplex system provided by Euroimmun^®^ AG (Luebeck, Germany).

Serum samples are collected routinely at UCLH during follow-up and stored. While we endeavoured to test sera for all patients who developed lupus psychosis, this was not always possible. For example, the patient may have had blood tests done at another hospital other than UCLH, they may not have been under UCLH follow-up at the time of psychosis, they may have refused at the time.

We used all the available sera; 10 of the 18 lupus psychosis patients in all. Sera from each available lupus psychosis patient were tested at two time points: the time of psychosis, and a paired sample one to five years later (depending on availability). Samples were individually matched for age, sex, ethnicity and time/date of the sample to two or three non-psychosis SLE controls (total controls, *n* = 27). They were aliquoted and frozen at –80°C according to standardised procedures. As described elsewhere [20], the commercially available and validated fixed cell-based assay (CBA) kit from Euroimmun^®^ was used for the detection of serum IgG antibodies binding to the following neuronal antigens: (i) NMDAR NR1/NR2b subunits; the VGKC-complex-associated proteins (ii) LGI1 and (iii) CASPR2; (iv) GABA_B_ Receptors B1 and B2; (v) AMPAR receptors type 1 and 2 and, finally; and (vi) DPPX. Five positive controls and one negative control were provided. At the time of the study, this kit did not include a non-transfected cell chip or AMPAR receptor antibody positive control. According to instructions provided, each mosaic was incubated for 30 min with a human serum at an initial 1:10 dilution, followed by a 0.2% PBS-tween wash for 5 min, and finally, incubation with the secondary antibody (flourescin-labelled goat anti-human IgG). Sample IgG binding to the surface of the transfected cells was revealed by green fluorescence and scored qualitatively (very strong positive, strong positive, positive, weakly positive, negative). For interpretation, four independent assessors (E.A., M.H., M.C., A.C.) scored each sample separately and blinded. Positive results were repeated to verify positivity, and to obtain a semi-quantitative measure of the antibody titre (1:10, 1:20, 1:40, 1:80, >1:80).

#### Live cell-based assays

All the samples were further tested in live cell-based assays for NMDAR and GABA_B_R antibodies by E.C. at the Department of Basic and Clinical Neuroscience, Institute of Psychiatry Psychology and Neuroscience, King’s College London, London, UK. The technique has been previously described [21, 22]. Briefly, human embryonic kidney (HEK) cells were transiently transfected with cDNA encoding our proteins of interest (GABA_B_receptor subunit‐1 and ‐2 or the NMDA Receptor subunit 1 and 2B). Live cells were incubated with the patient serum (1:20 or 1:100 for NMDAR and GABA_B_R, respectively) in DMEM supplemented with HEPES and 1% Bovine Serum Albumin (BSA) for 1 h at room temperature. Coverslips were then washed and fixed in 4% PFA. After further washes, they were incubated with secondary antibodies (1:1000; Alexa Fluor™ 568 goat anti-human IgG H&L; 1 h, room temperature) washed and mounted onto glass microscope slides with DAPI. Antibody binding to the expressed antigen was observed using a fluorescence microscope.

### Ethical approval

Permission to complete the clinical analysis was given by the Divisional Clinical Director for Medical Specialities at UCLH NHS Foundation Trust. Neuronal surface antibody tests were approved by the UCLH NHS Foundation Trust local Health Research Authority (HRA) Research Ethics Committee (REC), National Health Service (NHS), reference 16/SC/0494.

### Statistical analysis

Clinical data were analysed descriptively. Continuously distributed data were expressed as mean±SD. We used *t* tests to compare continuous variables and Fisher exact tests to compare categorical variables. Taking into consideration the small sample size of patients with this rare but important complication, *P*-values are to be interpreted with extreme caution. To control false discovery, a Bonferroni correction was utilised and a level of significance of *P* <0.002 denoted significance (critical *P*-value: 0.05, number of tests: 24). In this preliminary study, *P*-values are intended to be used conservatively and in an explorative manner. Data were analysed using STATA (version 15.1).

## Results

Of 709 patients with SLE, 18 (2.5%) were diagnosed with lupus psychosis (female:male ratio 5:1). There were no significant differences in mean age at diagnosis of SLE, duration of follow-up, gender or ethnicity between those who developed psychosis and those who did not. The mean time delay from the diagnosis of SLE to the diagnosis of psychosis was short (0.6 ± 2.9 years). Ten of the 18 patients developed lupus psychosis within 12 months of the diagnosis of SLE. Of the remaining patients, seven developed psychosis one to four years after the diagnosis of SLE, and the final patient unusually had their first psychotic episode nine years prior to the diagnosis of SLE.

There were no different clinical SLE features (as defined by ACR) between those who developed psychosis and those who did not (Table 2). Class III or greater World Health Organisation (WHO) lupus nephritis was only present in 3/18 (16.7%) of lupus psychosis patients, compared with 31.8% of those without psychosis (217/691). This difference was not significant (*P* =0.3). All lupus psychosis patients had at least one other neuropsychiatric manifestation in addition to psychosis. Although we were able to obtain only limited data on neuropsychiatric features in the SLE cohort without psychosis, the proportion of seizures between the two groups was not significantly different (*P*=0.167).

**Table 2:** Comparison of lupus psychosis patients (*n* = 18) *vs* without psychosis (*n* = 691)

		Lupus psychosis (*n* = 18)	SLE cohort (*n* = 691)	P-value
Age at diagnosis SLE (mean±SD) (years)		25.5 ± 9.7	29.1 ± 1.0.4	0.222^a^
Age at diagnosis psychosis (mean±SD) (years)		26.1 ± 9.4	NA	NA
Time delay SLE and psychosis (mean±SD) (years)		0.6 ± 2.9	NA	NA
Duration of follow-up (mean±SD) (years)		17.5 ± 11	14.1 ± 12.8	0.2648^a^
Gender, F: M, No (%)	Female:Male	5:1	10:1	NA
	Female	15 (83.3%)	633 (91.6%)	0.196
	Male	3 (16.7%)	58 (8.4%)	
Ethnicity, No (%)	Caucasian	11 (61.1%)	415 (60%)	0.908
	Afro-Caribbean	4 (22.2%)	152 (22%)	
	Asian	3 (16.7%)	76 (11%)	
	Chinese	0	27 (4%)	
	Other	0	21 (3%)	
Other SLE features, No (%)	Arthritis	17 (94.4%)	635 (91.9%)	1.000
	Rash (including cutaneous involvement)	14 (77.8%)	411 (59.5%)	0.146
	Vasculitis (e.g. skin, ophthalmic)	8 (44.4%)	Unknown	NA
	Serositis (pleuritis, pericarditis)	8 (44.4%)	254 (36.8%)	0.622
	Other autoimmune-associated disorder (Sjogren's, Raynaud's, Psoriasis)	8 (44.4%)	286 (41.3%)	0.812
	ITP/thrombocytopenia	4 (22.2%)	Unknown	NA
	Photosensitivity	4 (22.2%)	251 (36.4%)	0.320
	Alopecia	3 (16.7%)	160 (23.2%)	0.777
	Lupus nephritis	3 (16.7%)	217 (31.4%)	0.300
	Oral ulcers/mucocutaneous	3 (16.7%)	181 (26.2%)	0.585
NPSLE features, No (%)	Depression	11 (61.1%)	Unknown	NA
	Headache	6 (33.3%)	Unknown	NA
	Seizures	5 (27.8%)	123 (17.8%)	0.167
	Anxiety	3 (16.7%)	Unknown	NA
	Cognitive dysfunction	2 (11.1%)	Unknown	NA
	Hypomania	2 (11.1%)	Unknown	NA
	Visual disorder (e.g. maculopathy, loss of vision)	2 (11.1%)	Unknown	NA
Serological tests, No (%)	Anti-nuclear antibodies (ANA)	17 (94.4%)	673 (95.0%)	0.374
	Anti-RNP antibodies	9 (50.0%)	182 (26.5%)	0.033
	Low C3	9 (50.0%)	303 (43.8%)	0.637
	anti-dsDNA antibodies	8 (44.4%)	444 (64.2%)	0.133
	Low Hb	8 (44.4%)	Unknown	NA
	Anti-Ro antibodies	4 (22.2%)	248 (36.9%)	0.320
	Anti-Sm antibodies	4 (22.2%)	78 (13.0%)	0.144
	Anti-cardiolipin (G and M) antibodies	1 (5.6%)	196 (30.0%)	0.032
	Lupus anticoagulant (LAC)	1 (5.6%)	82 (14.0%)	0.710
	Anti-Ribosomal P antibodies	1 (5.6%)	Unknown	NA
	Rheumatoid factor (RF)	1 (5.6%)	160 (25.0%)	0.091
	Low lymphocyte	1 (5.6%)	550 (79.6%)	*P* <0.001
	Anti-La antibodies	1 (5.6%)	75 (13.0%)	0.710

Significance tests completed are Fisher’s exact (categorical) unless stated otherwise. ^a^*t* test. NA, not applicable. Bonferroni correction (*P* <0.002 denotes statistical significance)

In terms of serology, the majority of lupus psychosis patients tested positive for ANA (17/18, 94.4%), followed by anti-RNP antibodies (9/18, 50.0%) and anti-double-stranded DNA (8/18, 44.4%). Lupus psychosis patients may have more anti-RNP (50.0% *vs* 26.5%) and fewer anti-cardiolipin (5.6% *vs* 30.0%) antibodies, but these findings were not significant in our small sample with Bonferroni correction. Lupus psychosis patients had fewer instances of low lymphocyte count, but again, the sample is very small (5.6% *vs* 79.6%; *P* <0.001).

The distribution of the classification of reported psychotic symptoms is shown in Table 3. No negative symptoms of psychosis (as per ICD and DSM criteria) were reported. Investigations, treatments and outcomes in lupus psychosis are also shown in Table 3. Antipsychotic medication was used in 7/18 (38.9%), consisting of second-generation or atypical antipsychotic therapy with olanzapine (4/7), aripiprazole (2/7) and quetiapine (1/7). One patient required a combination of all of the following treatments: lithium, quetiapine, fluoxetine, venlafaxine, benzodiazepines and electro-convulsive therapy (ECT). In the long-term (one year onwards) management, 13/18 remained under follow-up at the time of the study. Of the five no longer under follow-up, three died (one of each of: adenocarcinoma aged 70, post-burns infection aged 32, bacterial endocarditis aged 49) and two were lost to follow-up (moved out of area).

**Table 3:** Clinical analysis of patients who developed lupus psychosis (*n* = 18) including psychotic manifestations, investigations, treatment and outcome of psychosis

Psychotic manifestations		No, %
Delusions	Paranoid	7 (36.8%)
	Grandiose	5 (26.3%)
	Depressive/nihilistic	1 (5.3%)
	Misidentification	2 (10.5%)
	Unknown	4 (21.2%)
Hallucinations	Auditory	12 (57.1%)
	Visual	8 (38.1%)
	Olfactory	1 (4.8%)
	Other/unknown	0
Thought disorder		3 (14.3%)
Lack of insight		5 (23.8%)
Investigations		No, %
Imaging	Abnormal EEG	5 (27.8%)
	Abnormal MRI	4 (22.2%)
	Abnormal brain perfusion scan	3 (16.7%)
	Unknown	6 (33.3%)
Cerebrospinal fluid (CSF) examination	Normal CSF	5 (27.8%)
	Abnormal CSF	1 (5.6%)
	Unknown	12 (66.7%)
Treatment (psychiatric and other)		No, %
Immunosuppressive: Induction therapy	IV/IM methylprednisolone	13 (72.2%)
	Cyclophosphamide	10 (55.6%)
	Prednisolone (high)	7 (38.9%)
	Plasma exchange	5 (27.8%)
	Azathioprine	4 (22.2%)
	Prednisolone (med)	2 (11.1%)
	Rituximab	3 (16.7%)
	Unknown	0
Immunosuppressive: Maintenance therapy	Prednisolone (low)	16 (88.9%)
	Azathioprine	9 (50.0%)
	Hydroxychloroquine	5 (27.8%)
	Mycophenolate mofetil	2 (11.1%)
	Prednisolone (high)	1 (5.6%)
	Methotrexate	1 (5.6%)
	Unknown	0
Psychiatric treatment	Antipsychotic	7 (38.9%)
	Antidepressant	7 (38.9%)
	Benzodiazepine	5 (27.8%)
	Mood stabiliser	1 (5.6%)
	Electro-convulsive therapy (ECT)	1 (5.6%)
	Unknown	2 (11.1%)
Psychiatric outcome		No, %
Short term outcome: in reference to the 6-month period after the psychotic episode	Resolution (<1 month)	12 (66.7%)
	>1month duration of symptoms	3 (16.7%)
	Partial remission with residual symptoms	3 (16.7%)
	Unknown	0
Long-term outcome: in reference to the course of psychotic illness 12 months after the initial episode	Full remission nil further psychosis	12 (66.7%)
	1 further episode	2 (11.1%)
	2 further episodes	1 (5.6%)
	3 further episodes	1 (5.6%)
	4 or more further episodes	1 (5.6%)
	Unknown	1 (5.6%)

Prednisolone doses: (0–7.5 mg/day), medium (7.5–19mg/day) and high (≥20mg/day).

**Fig. 1:**
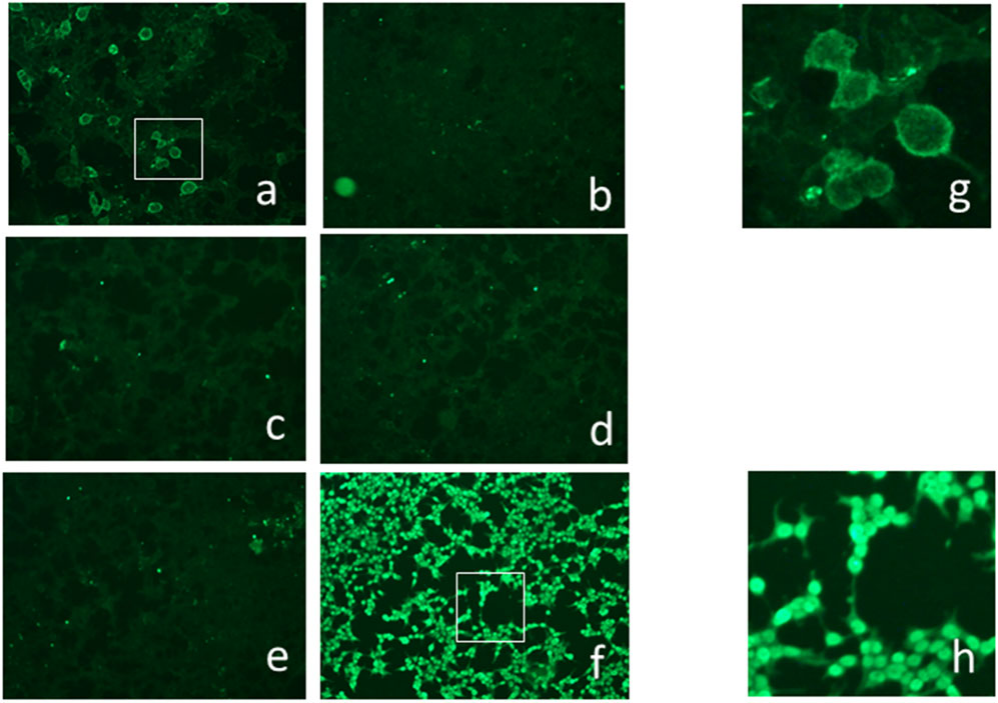
Lupus psychosis sample showing GABA_B_R positivity on fixed cell-based assay Immunostaining of commercial cell-based assay (**a**–**f**) showing HEK cells expressing: (**a**) GABA_B_R (R1/R2), (**b**) DPPX, (**c**) LGI1, (**d**) AMPAR1/2, (**e**) CASPR2, (**f**) NMDAR. Sera were diluted 1:10 and antibody binding visualised with goat anti-human IgG. Note the surface binding of the GABA_B_R (**a**) and the nuclear staining of the NMDAR cells, which are strongly permeabilised (**f**), and seen in all ANA-positive SLE patients (**g**, **h**). Higher magnifications of GABA_B_R and NMDAR expressing cells, taken from **a**, **f**. AMPA: alpha-amino-3-hydroxy-5-methyl-4-isoxazolepropionic acid; CASPR2: contactin associated protein 2; DPPX: dipeptidyl aminopeptidase-like protein 6; GABA: gamma-Aminobutyric acid; HEK: human epithelial kidney; LGI1: leucine-rich glioma inactivated 1; NMDA: N-methyl-D-aspartate; SLE: systemic lupus erythematosus.

GABA_B_R antibodies were positive in 3/10 (30%) psychosis cases (patients 1-3, full characteristics of these three patients are shown in Table 4) and 3/27 controls (11.1%) (OR = 3.43, 95% CI 0.36–30.68, *P* =0.1143). Fig. 1 shows an example of GABA_B_R autoantibody in the serum of ‘patient 1’. [Table keab160-T4] There was no other antibody positivity found of neuronal surface antibodies tested. Of lupus psychosis patients positive for GABA_B_R antibodies at either time point, one was positive at the time of psychosis only and one was positive on the later sample only, but notably had two further psychotic episodes during follow-up. The final patient was antibody positive on both the sample taken at the time of psychosis and persistently positive three years later. All GABA_B_R autoantibody positive samples were also reproducibly positive on repeated fixed cell-based assay testing using the same methodology, and became demonstrably weaker on diluting the serum providing titres ranging from 1:10 to >1:80. Due to the different fixation method used for the NMDAR autoantibody, these cells were permeable to ANA and all SLE psychosis samples and ANA-positive controls showed intense antibody binding to the nuclei, but not to the cell membrane where the NMDAR was expressed (Fig. 1). Using live cell-based assays, however, that do not detect antibodies to intracellular components, there was no evidence of any binding to GABA_B_R and also confirmed negative to NMDAR.

**Table 4 keab160-T4:** Characteristics of GABA_B_R autoantibody positive lupus psychosis patients on fixed cell-base assay (*n* = 3)

	Patient 1	Patient 2	Patient 3
Sex/ethnicity	F/AC	M/C	F/C
Follow-up (years)	18	30	12
Age at diagnosis SLE/psychosis	35/35	16/16	31/31
Total no of psychosis episodes	1	1	3
Psychotic manifestations	Delusions (not specified), auditory hallucinations	Manic episode with grandiose delusions and change in personality. Grandiose beliefs about self. Persecutory delusions and delusions of misidentification.	Mania with grandiose delusions, visual hallucinations and change in behaviour.
Other NP features	Seizures	Depression, headache	Headache (frontal)
Non-NP SLE features	Arthritis, pleuritis, lupus nephritis (class 3 or greater on WHO criteria)	Rash, arthritis, interstitial lung disease, Raynaud’s	Rash, arthritis, fatigue, serositis, Raynaud’s
CNS investigations	Normal MRI, abnormal EEG	Normal MRI, nil other results	EEG normal, normal MRI, normal LP (HSV -ve, oligoclonal band -ve)
Induction therapy	IV methylprednisolone, cyclophosphamide, prednisolone (high)	Azathioprine, prednisolone (med)	IV methylprednisolone, cyclophosphamide
Maintenance therapy	Prednisolone (low), azathioprine	Azathioprine, prednisolone (low)	Prednisolone (high)
Psychiatric medication	Nil psychotropic, acute only with haloperidol, lorazepam)	Olanzapine	Olanzapine, diazepam
Long-term outcome of psychosis	Resolution after 1 week. No recurrence, died (bacterial endocarditis) aged 49.	Good response to immunotherapy and olanzapine (20 mg), no recurrence, long-term depression requiring treatment (sertraline). Alive and under follow-up.	First manic episode resulted in 1 week admission, followed by resolution. Second episode 2 years later (improved with IV methylprednisolone pulses after 5-6 days). Third admission with similar presentation. Olanzapine and diazepam used and repeated pulses cyclophosphamide. Continues on long-term olanzapine. Alive and under follow-up.
First serum sample (at time of psychosis)	GABA_B_R (1:80)	GABA_B_R (1:20)	Negative all
Second serum sample (at least 1 year later)	Negative all	GABA_B_R (1:20)	GABA_B_R (1:20)

Prednisolone doses: (0–7.5 mg/day), medium (7.5–19mg/day) and high (≥20mg/day). AC: Afro-Caribbean; C: Caucasian; F: female; M: male; NP: neuropsychiatric; SLE: systemic lupus erythematosus.

## Discussion and conclusion

We present the longest recorded follow-up study of a well-characterised clinical cohort of patients with lupus psychosis from a single specialist centre. This cohort was first reported in 2008 [16]. The considerably extended duration of follow-up (14.1 ± 12.8 years) is much longer than existing multicentre studies [e.g. mean 7.4 ± 4.5 years, Systemic Lupus International Collaborating (SLICC) group [23]]. We have also added a more detailed characterisation of psychotic symptoms, and additional anti-neuronal antibody tests. These data extend some of our existing knowledge of this rare complication, and provide preliminary data to suggest further potential avenues for exploration.

Our study confirms that lupus psychosis is a rare complication of SLE (2.5%). Similar to other NPSLE phenomena, the prevalence of lupus psychosis varies wildly in existing studies depending on the use of the ACR case definition (from 0% to 29.8% [7, 8]). However, our result is in agreement with other studies that use a strict criteria for lupus psychosis; for example, the SLICC cohort reported a prevalence of 1.53% in 1826 patents with mean 7.4 years of follow-up [23].

As previously established, we confirmed that lupus psychosis is usually an early complication with just over half (10/18, 55.6%) developing psychosis concurrently with diagnosis of SLE [7, 8, 16, 24]. It is a complication that requires early aggressive treatment, and is followed by long-term remission in the majority of cases [25, 26]. Lupus psychosis frequently occurs in association with additional neuropsychiatric manifestations (e.g. seizure and/or depression in a third), as shown by other groups [27]. Our gender ratio suggests that a higher proportion of males develop lupus psychosis than would be expected by the overall female to male ratio in SLE (F:M in lupus psychosis 5:1, F:M in all SLE is 10:1). While this is supported by previously published data [23, 28], the difference was not significant in our relatively small sample.

In terms of psychosis symptoms, the majority of presentations included paranoid and grandiose delusions, as well as auditory and visual hallucinations. This is in keeping with previous literature on organic psychosis [8, 29], but adds to the psychiatric phenomenology in lupus psychosis which is often not described in detail [30]. The finding of a higher proportion of anti-RNP antibodies (*P* =0.033) and lower proportion of anti-cardiolipin antibodies (*P* =0.032) is in contrast to recent meta-analysis of NPSLE, but may be explained by the suggestion that ‘focal’ NPSLE such as stroke is more likely to be related to thrombogenic antibodies than ‘non-focal’ NPSLE such as psychosis [12, 31]. This highlights a need to look at NPSLE complications in isolation.

Our results suggest that GABA_B_R antibodies may be more frequently found in lupus psychosis using the commercially available fixed cell-based assay. However, this needs to be interpreted with caution as positivity was not statistically significant in our small sample and, moreover, it was not replicated on the live cell-based assay. The levels of the antibodies titre were also low (e.g., 1:20). It is also possible that this was a chance finding due to higher levels of immunosuppression in the matched controls, as perhaps suggested by the higher prevalence of proliferative nephritis. This may also explain the lower lymphocyte count found in SLE patients who did not have psychosis. However, it is not possible to draw definitive conclusions from this sample and this result remains exploratory.

Antibodies binding to short peptides of the GABA_B_R have been previously identified in patients with NPSLE, including two patients with lupus psychosis, using ELISA [32]. GABA_B_R antibodies were first identified by a cell-based assay, similar to that used here, in limbic encephalitis [33] and GABA_B_R system dysfunction has been implicated in post-mortem studies in schizophrenia [34]. If implicated, the concurrent positivity for GABA_B_R in SLE controls suggests that other mechanisms, which may be SLE specific, are important in the development of lupus psychosis. The lack of NMDAR surface antibodies in lupus psychosis on both live or fixed cell-based assays is surprising, as NR2 has been reported to be a target of antibodies in NPSLE [15, 35] and NMDAR antibodies (usually the NR1 subunit) have been identified in first-episode psychosis without SLE [36, 37]. It is possible that rather than a candidate brain-specific autoantibody or biomarker, other mechanisms could cause lupus psychosis, including innate immunological and cytokine mediated effects and this requires further study.

Our study has a number of limitations. Firstly, despite the fact that the cohort had undergone a lengthy and detailed follow-up at a single specialist centre, lupus psychosis is a rare complication and our sample size is small. While our findings may point towards important targets for further research, they are exploratory and must be interpreted tentatively. In addition, while efforts were made to retrieve historic information, this was not always possible. Psychiatric information was relatively scarce and validated rating scales for psychotic symptoms (e.g. Positive and Negative Syndrome Scale, PANSS) rarely utilised. Additionally, as psychosis is a very early complication of SLE—sometimes occurring prior to formal diagnosis—we do not have information on the nature of any prodromal or initial symptoms that pre-dated the formal diagnosis of SLE in this cohort. In terms of biomarker testing, we used a commercially available fixed cell-based assay kit for the detection of antibodies, which has methodological superiority to peptide ELISA, but permeability of the fixed NMDAR transfected cell to ANA antibodies can make interpretation challenging to inexperienced groups. Samples required verification on live cell-based assays, which measure only those antibodies binding to the cell surface antigen but use a higher dilution with lower sensitivity. The study would benefit from other centres attempting to replicate the findings on their own archived samples.

To conclude, our study of a large cohort of patients with SLE followed up for a mean of 14.1 years demonstrates that lupus psychosis is a rare and early complication of SLE, with a good prognosis. There is an urgent need for more comprehensive psychiatric evaluation of patients with lupus psychosis. This preliminary study demonstrates that more work is needed to identify potentially pathogenic biomarkers in SLE and psychosis, which may be immunotherapy responsive.
